# Experimental Characterization and Evaluation of Crude Spiking Influence on Oil/Water Dispersed Flow in Pipe

**DOI:** 10.3390/molecules28176363

**Published:** 2023-08-31

**Authors:** Hamidreza Asaadian, Milan Stanko

**Affiliations:** Department of Geoscience and Petroleum, Norwegian University of Science and Technology, S. P. Andersens veg 15, 7031 Trondheim, Norway; milan.stanko@ntnu.no

**Keywords:** oil/water emulsion, crude spiking, emulsion characteristics, rheology model, mini-loop setup, droplet size distribution

## Abstract

This study centers around examining the impact of introducing varying (small) quantities of crude oil into mineral oil (Exxsol D60) on the resultant properties of dispersions and emulsions in oil–salty-water mixture properties such as rheology, droplet size distribution, separation duration, and interfacial tension. The experimentation encompassed bottle tests and a compact flow loop configuration featuring a 2 m horizontal pipe segment. The findings indicate that blends of oil infused with crude oil, combined with salty water at water ratios of 25% and 50%, necessitate an extended duration for separation and for the establishment and stabilization of interfaces, in contrast to mixtures of unaltered oil and saline water. To illustrate, in samples with spiking concentrations ranging from 200 to 800 ppm within a 25% water fraction, the separation period escalates from 51 s to 2 min and 21 s. Interestingly, when the water fraction increased to 75 percent, the impact of crude oil spiking on separation time was minimal. The analysis revealed that the Pal and Rhodes emulsion viscosity model yielded the most accurate predictions for the viscosity of resulting emulsions. The introduction of crude oil spiking elevated emulsion viscosity while diminishing interfacial tension from 30.8 to 27.6 mN/m (800 ppm spiking). Lastly, a comparative assessment was performed between droplet size distributions in the devised dispersed pipe flow and observed in an actual emulsion system comprising crude and salty water.

## 1. Introduction

Liquid–liquid dispersions in pipelines are common in industrial processes like oil and gas transport [1]. Emulsions of oil–water affect equipment performance and flow assurance negatively due to issues like corrosion and pressure drop [2]. Therefore, preventing emulsion formation is crucial [3,4]. Modeling such dispersions in oil production often lacks reliability, requiring experiments. Oil–water emulsions, like oil-in-water (OiW) or water-in-oil (WiO) [5,6], are stabilized by surfactants at the interface, with micron-sized droplets. Crude oils consist of diverse components, categorized as saturates, aromatics, resins, and asphaltenes (SARA fractions), impacting physicochemical properties. Oil density, viscosity, and components like asphaltenes affect dispersion characteristics [7,8,9].

**Figure 1:**
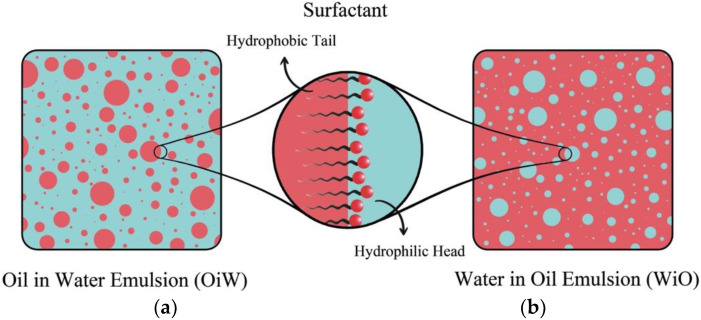
Schematic of emulsion structures. (**a**) OiW emulsion; (**b**) WiO emulsion.

The viscosity and density of oils affect the characteristics of oil–water dispersions [10,11,12,13,14]. Also, asphaltenes (high-molecular-weight polar components) are abundant in crude oils, particularly heavy oils, and function as natural emulsifiers (Figure 1). Resins, fatty acids such as naphthenic acids, porphyrins, wax crystals, and other crude oil components are also surface active, although they cannot generate stable emulsions on their own most of the time [15,16,17,18]. Asphaltenes in oil are solubilized by resins, which remove them from the interface, decreasing emulsion stability [19,20].

Oil production is hindered by surface-active components, causing flow assurance issues. Emulsion stability affects droplet dispersal and pressure drop due to interfacial behavior. Current research has focused on oil–water emulsion stability [21,22,23]. Consequently, in most investigations, synthetic/mineral oils replace crude in experiments for cost and safety reasons. However, mineral oils often cannot produce stable emulsions with water on their own. Therefore, separation tests carried out using model oils are often more efficient than those conducted with real crudes, according to the literature [24,25]. To make the test oil–water mixture more representative, an appropriate surfactant must be chosen to generate and stabilize emulsions. Prior to the pipeline flow testing, certain emulsion parameters, such as droplet sizes, stability, and rheological properties at specified temperatures, are typically studied in beaker tests [26,27].

Example of stabilizers that are added to mineral oils are oil-soluble surfactants and hydrophobic nanoparticles. Table 1 Shows the previous studies of stabilizers examples.

This study delves into the synthesis of an artificial oil–water mixture. This is achieved through the introduction of crude oil into a mineral oil substrate (Exxsol D60), a process termed “crude spiking.” By leveraging the inherent surfactant properties of the original crude oil, we aim to generate emulsion properties akin to the natural fluid system. This innovation could potentially curtail the need for expensive test campaigns within industrial setups and propose an environmentally friendly method of investigation.

Furthermore, we explore the impact of crude spiking on the physicochemical attributes of the emulsion system, the rheological behavior of the resulting oil–water dispersion, and its interplay with droplet size distribution. Emulsion viscosity and droplet size distribution are assessed under ambient conditions, which are considered constraints on the work. On the other hand, the advantage of this study lies in achieving desired stability in emulsions solely through basic components.

## 2. Materials and Method

### 2.1. Emulsion Preparation

The following fluids were used to investigate the effect of spiked crude on oil–water dispersion flow: mineral oil Exxsol D60 (μ = 1.31 cP, ρ = 787.45 kg/m^3^ @ 15 °C), distilled water with added wt% 3.4 NaCl (μ = 0.99 cP, ρ = 1020.75 kg/m^3^ @ 15 °C), and crude oil (Table 2). Crude properties at 15 °C are given in Table 3. Model fluids were prepared by different spiking amounts of crude oil with Exxsol D60. These amounts vary from 100 to 800 ppm.

Additionally, test data using four different crude oils were taken from the work of Plasencia et al. [34] and compared against the data gathered in this work. The properties of these crude oils are shown in Table 4. All the performed experiments by Plasencia et al. [34] were of oil/water dispersed flow in the pipe at 60 or 70 °C.

### 2.2. Test Mini-Flow Loop

**Figure 2:**
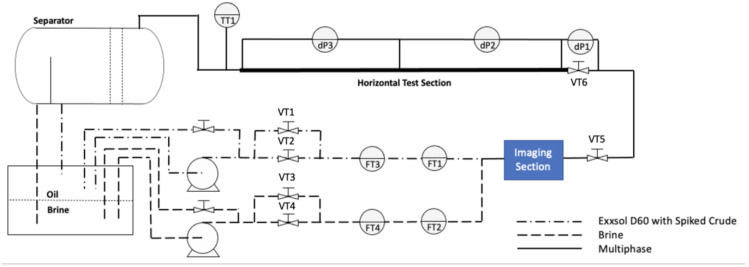
Layout of test mini-flow loop.

The experiments were performed in the mini-loop setup of the SINTEF Multiphase Flow Lab in Tiller, Norway. A detailed sketch of the test section is shown in Figure 2. The 2 m long horizontal test section with an inner diameter of 8 mm is made of stainless-steel and fully insulated. The test section was horizontally aligned at 0 ± 0.1°. A 1 m^3^ storage tank was originally filled with equal amounts of distilled water with 3.4 wt% NaCl, and Exxsol D60. Liquids are pumped from the tank separately before being mixed at the test section inlet. Centrifugal pumps are used to circulate the liquids. The flowrate of each liquid was measured before the mixing section. Also, valves were installed to control the system’s overall pressure (VT1, VT2, VT3 and VT4) before the liquids entered the test section. Likewise, a manual choke valve (VT6) at the upstream of the pre-separator was used to control the liquid level in the vessel. An imaging section was placed after the mixing section to take images from dispersed flow for further droplet size distribution study. It should be noted that all flow conditions of oil and water in these experiments exhibited a fully dispersed flow pattern.

During mini-loop setup experiments, 100 test points were investigated by studying 4 different mixture velocities, 17 oil volume fractions and 9 crude spiking concentrations. The ultimate combinations tested are presented in the test matrix shown in Table 5.

### 2.3. Imaging Section (CANTY Particle Sizing Analyzer)

Photos of the emulsions (droplets) were taken by a CANTY particle sizing analyzer. The CANTY particle sizing analyzer is designed to allow the user to study fluids under a variety of pressures, temperatures, and flow rates. When used in combination with the Canty Vision Client TM Software (version 1), it delivers sample or continuous, microscopic, non-destructive viewing. Precision optics are used in the vision system, which has integrated illumination, to enhance the image before it is shown.

A high-resolution/high-speed CCD/CMOS image sensor is used in conjunction with a microscopic lens system. Zoom and focus capabilities, variable illumination, and numerous objective lens packages to accommodate a range of sizes are all included in the system. A FUSEVIEW glass pane works as a product contact barrier in CANTY particle size systems. All droplets are counted and measured manually using ImageJ software (version 1.54f) to determine droplet-size-distribution curves for each flow condition.

### 2.4. Separation Time Using Bottle Tests with Spiked Exxsol D60 and Water

Bottle shake tests were conducted at ambient temperature (23 °C) with oil–water mixtures. The mixtures were stirred at 750 rpm for 30 s using a magnetic stirrer and were then allowed to separate. The time required for separation was then recorded. The tests were conducted with distilled water containing 3.4 wt% NaCl and Exxsol D60 spiked with different amounts of crude oil (200, 300, 400, 500, 600, 700, and 800 of ppm in mass basis). A solution with 800 ppm spiking concentration was made initially and then diluted by adding pure Exxsol D60 to obtain other solutions with lower concentrations. Several water volumetric fractions (hereafter referred to as water cut, i.e., WC) were used: 25%, 50%, and 75%. Table 6 summarizes the conditions for bottle separation tests.

**Figure 3:**
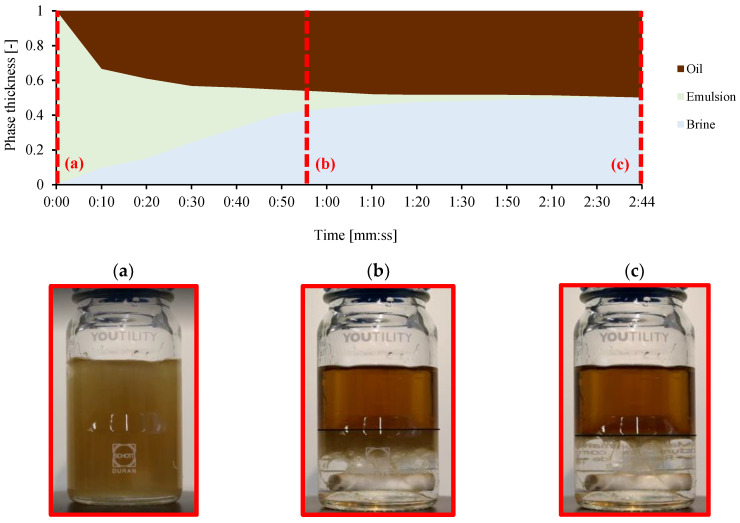
Separation bottle depicting Exxsol D60 with 300 ppm crude oil, WC 50%—conditions of test bottle at (**a**) starting point of separation, (**b**) t_in_ and (**c**) t_sep_.

The separation process was filmed three times for each sample and the video was analyzed afterwards. Two different separation times are reported, counted after the magnetic stirrer stops (Figure 3a): t_in_ is the time it takes for the interface between oil and water to stabilize at a specific height, as shown in Figure 3b. The black line is the interface of clean oil, and the dark-brown layer between clean oil and transparent water at the bottom is an emulsion layer. t_sep_ is the time until there is complete phase separation. Separation is complete when the mixture has returned to its original two pure phases, as shown in Figure 3c. Here, the black line is the interface of clean oil at the top and transparent water at the bottom.

**Figure 4:**
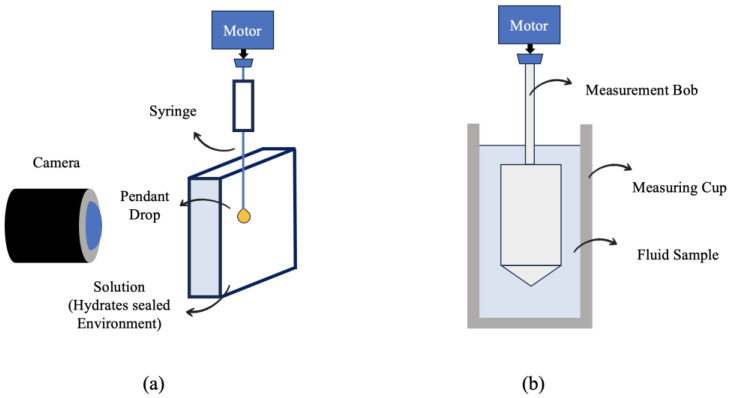
Schematic of (**a**) pendant drop and (**b**) rheometer setups.

An Anton Paar Physica MCR 301 rheometer was used to measure the fluids’ rheological properties. All the investigations were carried out with a concentric cylinder shape (CC27). During the measurements, Peltier elements were used to keep the temperature at the desired level. The fluids’ rheological properties were investigated at various shear rates (s^−1^). A Spectro-densitometer was also used to determine the density of the fluids. The KSV pendent drop was employed to measure interfacial tensions (Figure 4).

## 3. Results and Discussion

### 3.1. Density, Viscosity, and Interfacial Tension

**Figure 5:**
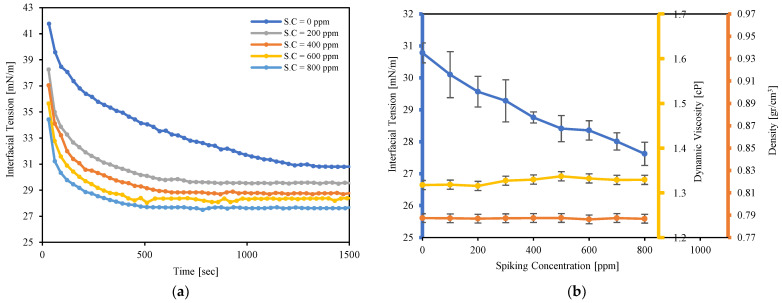
Effect of crude spiking concentration on Exxsol D60 properties: (**a**) Interfacial tension over time, (**b**) Density, Viscosity, and Interfacial tension.

Viscosity, density, and interfacial tension of Exxsol D60 with different amounts of crude spiking in the presence of salt water are measured and reported in this subsection. Figure 5a compares interfacial tension between Exxsol D60 and salt water for various concentrations of crude spiking over time. When crude oil and brine first came into contact, the interfacial tension between them was at its highest level. Over time, however, it gradually decreased, to reach equilibrium. This indicates that over time, the crude oil’s surface-active molecules are being adsorbed into the interface. Adding crude spiking results in lower interfacial tension and it helps earlier equilibrium in interfacial tension during pendent drop experiments. For example, adding crude spiking up to 800 ppm into pure Exxsol D60 brings down its interfacial tension from 30.8 to 27.6 mN/m. As is visible in Figure 5a, interfacial tension of a droplet of pure Exxsol D60 in salt water stabilized after approximately 1300 s; meanwhile, stabilization time for Exxsol D60 with 800 ppm spiking is roughly 510 s. This is because surface-active components like asphaltenes, resins, fatty acids, and wax crystals present in the crude move toward the droplet surface and affect the surface forces. The main mechanism causing a quicker gradual decline in the IFT with respect to time is the concentration gradient of surface-active material between the oil bulk and the interface. Figure 5b shows the behavior of interfacial tension, oil density and viscosity versus spiking concentration. The density is nearly constant, but a smooth rise is noticeable in the dynamic viscosity of model oils by increasing the spiking concentration from 1.31 to 1.33 cP.

**Figure 6:**
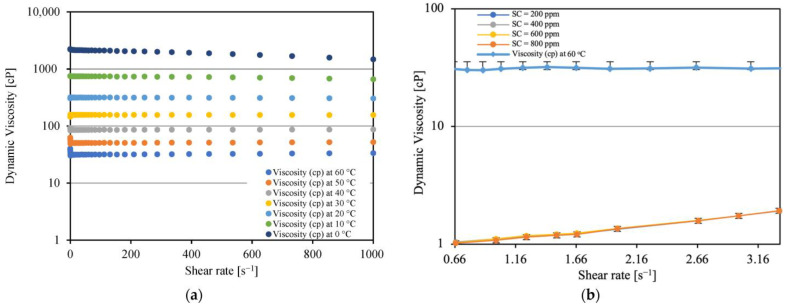
Viscosity vs. shear rate (**a**) of crude oil for different temperatures and (**b**) Exxsol D60 with various concentrations of crude spiking.

Figure 6a presents a variation of dynamic viscosity of crude oil versus shear rate from 0.1 to 1000 (s^−1^) at different temperatures, from 0 to 60 °C. The viscosity reduces as the temperature rises. For temperatures higher than 20 °C, the viscosity of the fluids is practically constant as a function of the shear rate, showing that the fluids have Newtonian behavior. When the temperature falls below 20 °C, the crude oil exhibits shear-thinning behavior at low shear rates. In Figure 6b, the viscosities of the spiked model oil at 25 °C and crude oil at 60 °C were compared at low shear rates (from 0.66 to 3.50 s^−1^). For the spiked model oils, the viscosity increases as a function of the shear rate (shear-thickening behavior). There was no noticeable effect by change of the spiking concentration. The crude oil viscosity is nearly constant with the shear rate.

### 3.2. Inversion Point

**Figure 7:**
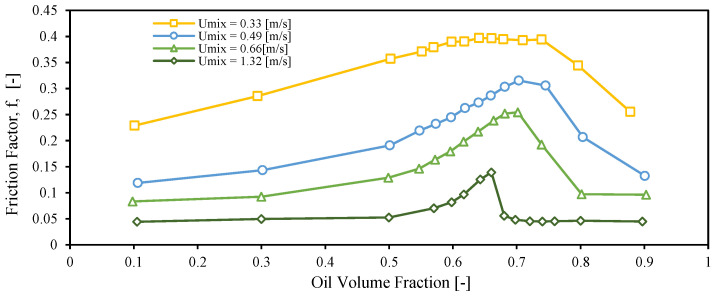
Effect of mixture velocity on inversion point with 400 ppm spiking concentration.

Figure 7 depicts variations in friction factor over different oil volume fractions at four distinct mixture velocities, using spiked oil with 400 ppm of crude. The friction factors have been calculated from the values of pressure drop measured along the horizontal test section of the mini-loop set-up, with the following equation:

(1) $$f=\frac{2\tau_{w}}{r\cdot V^{2}}=\left(\frac{DP}{L}\right)\frac{2D}{r_{m}U_{m}^{2}},$$

*D*, *L* and τ*_w_* denote the pipe length, inner diameter, and shear stress on the inner wall of the pipe, respectively. *DP* represents the pressure drop along the specific pipe length. The velocity used is the mixture velocity. According to the equation, increases in mixing velocity result in a decreased friction factor.

For a fixed mixture velocity, the friction factor exhibits a typical emulsion behavior with respect to oil volume fraction, i.e., there is an increase and posterior decrease with increasing values of oil volume fraction. The inversion point (where the continuous phase changes from water to oil) is shifted to higher oil volume fractions as the mixture velocity decreases. The inversion “peak” becomes sharper by raising the mixture velocity. The oil volume fractions where the inversion point occurs for four investigated mixture velocities are presented in Table 7.

**Figure 8:**
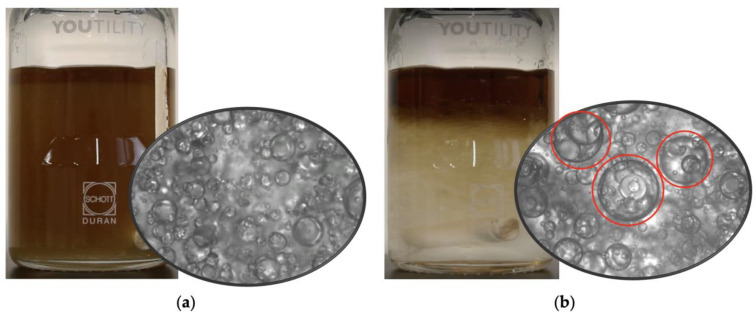
Exxsol D60 (+400 ppm crude spiking)—salty-water emulsions, (**a**) before and (**b**) after inversion point.

The structure of the emulsions in the vicinity of the inversion point was studied by preparing bottle samples at the same water cuts studied in the mini-loop setup and taking photos. Figure 8 shows images of emulsions of Exxsol D60 spiked with 400 ppm crude oil and water collected before (a) and after (b) the occurrence of inversion. These pictures were taken right after the preparation and blending. The sample obtained at a water cut below the inversion point shows a homogeneous, stable, brown mixture (Figure 8a). The microscopic image confirms that there are water droplets in a continuous oil phase.

The sample taken at a water cut above the inversion shows a continuous unstable water mixture (Figure 8b). The microscopic image shows that water-in-oil emulsion droplets are present in a continuous water phase (multiple emulsion). The water-in-oil emulsion droplets appeared to immediately begin to coalesce under quiescent conditions, while salt water accumulated at the bottom of the bottle. Similar outcomes were seen for the rest of the spiking concentrations.

**Figure 9:**
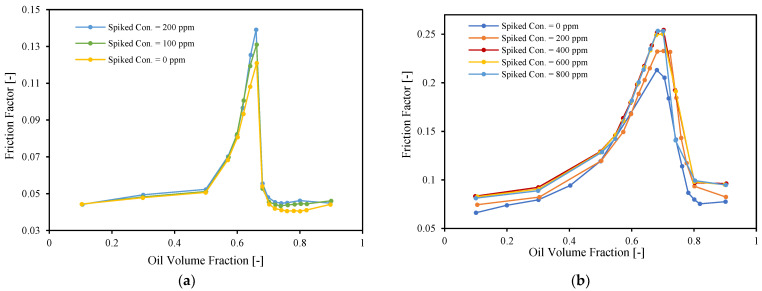
Effect of spiking concentration and mixture velocity on friction factor, (**a**) U_mix_ = 1.32 [m/s], (**b**) U_mix_ = 0.66 [m/s].

The effects of both spiking concentration and mixture velocity on the friction factor are shown in Figure 9. Figure 9a shows the friction factor versus the oil volume fraction for a mixture velocity equal to 1.32 m/s and 2 spiking concentrations. The friction factor increases with an increase in the spiking concentration for the continuous water region.

Figure 9b shows the friction factor versus the oil volume fraction for a mixture velocity equal to 0.66 m/s and 4 spiking concentrations. As with Figure 9a, the friction factor somewhat increases with an increase in the spiking concentration, but not for all water cuts. The differences between the three highest spiking concentrations are negligible. This could be because at lower mixture velocities the oil and water tend to separate and the assumption of dispersed flow (and the use of an effective viscosity and friction factor) is not valid anymore.

### 3.3. Emulsion Viscosity

Emulsions often behave like Newtonian fluids at low dispersed-phase fractions. However, depending on the flow hydrodynamics and shear-rate field, they can behave like non-Newtonian fluids at high dispersed-phase fractions. Besides the concentration of the dispersed phase, other factors can affect the rheological behavior of the oil/water emulsion [18], such as continuous phase viscosity (𝜇_𝑐_), temperature (𝑇), the density of the continuous phase (𝜌_c_), shear rate (𝛾), dispersed-phase viscosity (𝜇_𝑑_), droplet size distribution (𝑑), the density of dispersed phase (𝜌_𝑑_), and emulsifying agents and their concentration.

Emulsion rheology behavior can be characterized using the term relative viscosity (𝜂_𝑟_), which is known as the ratio between the effective viscosity of the emulsion (𝜂_e_) and continuous-phase viscosity (𝜇_𝑐_). Ω represents dispersed volume fraction. Table 8 presents a few rheology models for oil–water emulsions that are developed and presented in the literature [35,36,37,38,39].

**Figure 10:**
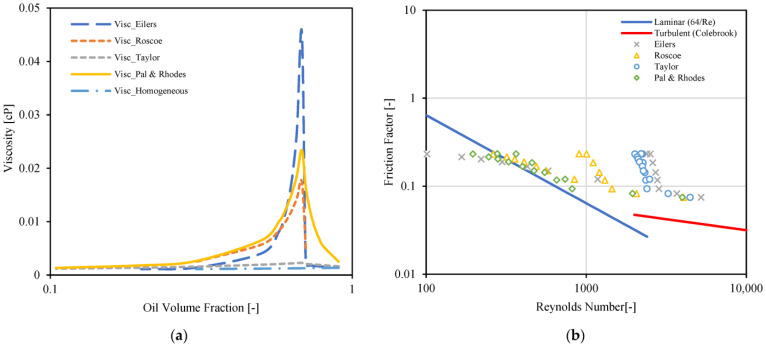
(**a**) Emulsion viscosity prediction by rheology models, (**b**) Interpretation of laminar and turbulent flow points on friction factor vs. Reynolds number plot.

Figure 10a presents values of mixture viscosity versus oil volume fraction for four emulsion viscosity models. Three of these models, namely Eilers [36] Roscoe [37] and Pal and Rhodes [39] predict an inversion point at around 70% oil volume fraction. The Pal and Rhodes [39] and Eilers [36] models output the highest values of viscosity when compared to the rest.

In Figure 10b, four emulsion viscosity models are studied to try to find the most suitable for the current dispersed oil–water system. The procedure is as follows: Reynolds numbers are computed for each experimental point, using experimental values and the effective viscosity (𝜇*_eff_*) output from the emulsion viscosity models. The friction factor is calculated from experimentally recorded pressure drops (as explained earlier, using Equation (1) [34]). The corresponding Reynolds numbers and friction factor values are then plotted for the four emulsion viscosity models studied (markers in Figure 10). The friction factor equations for the laminar regime (Equation (2)) and the turbulent regime (Colebrook) (Equation (3)) are also plotted in the figure with continuous lines.

*f* = 64/*Re*, (2)

(3) 1f1/2=2log eD3.7+2.51Re f1/2,

where *e* and *D* are the pipe roughness (0.045 mm) and internal diameter, respectively. The most suitable emulsion viscosity model will output values that lie closest to the turbulent and laminar lines. In this case, this corresponds to the Pal and Rhodes [39] model. This model is then selected for further studies.

**Figure 11:**
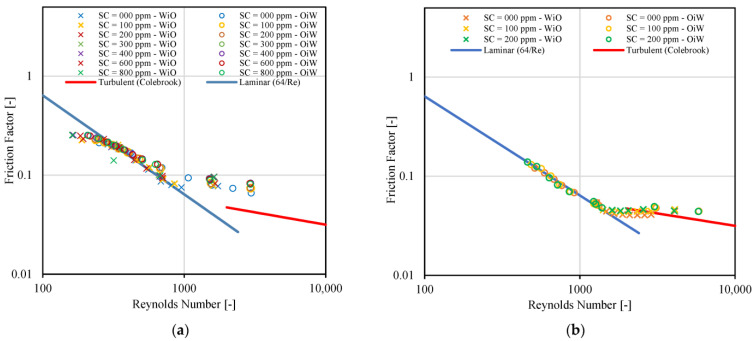
Interpretation of laminar and turbulent flow points on friction factor vs. Reynolds number plot, (**a**) U_mix_ = 1.32 [m/s], (**b**) U_mix_ = 0.66 [m/s].

A similar process to the one used for Figure 10b is used to generate Figure 11a, but using the Pal and Rhodes model only. The constants (K_PR_) of the Pal and Rhodes model [39] were tuned to improve the match (≈0.89). This was carried out manually and by visual inspection. The points depicted in the figure are for several values of crude spiking, emulsion type (OiW and WiO) and for a mixture velocity equal to 0.66 m/s. Most of the points are in the laminar regime. The points that are close to the turbulent region are in the transitional regime. Figure 11b is similar to Figure 11a, but for a mixture velocity equal to 1.23 m/s. In this case, more points are in the transitional regime and in the turbulent regime. The constants of the Pål and Rhodes model [39] were tuned to improve the match (≈0.86).

**Figure 12:**
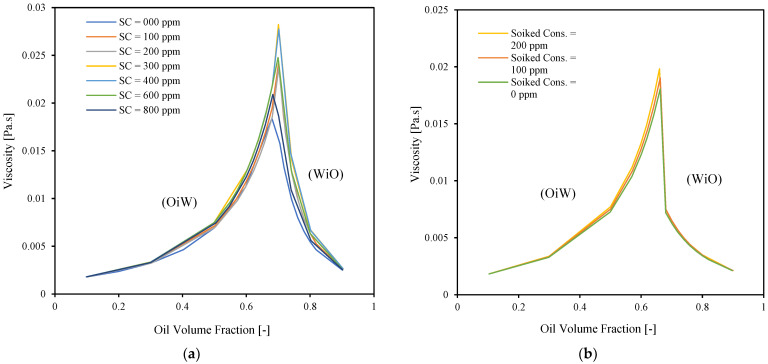
Predicted viscosities based on Pal and Rhodes model for (**a**) U_mix_ = 1.32 [m/s], (**b**) U_mix_ = 0.66 [m/s].

Predicted viscosities based on the tuned Pal and Rhodes model [39] are presented in Figure 12 for two mixture velocities: 0.66 and 1.23 (m/s). A similar trend to the one shown in Figure 9 is observed: the viscosity-changing trend with spiking concentration is monotonic in high mixture velocity, while it is non-monotonic at low mixture velocity.

**Figure 13:**
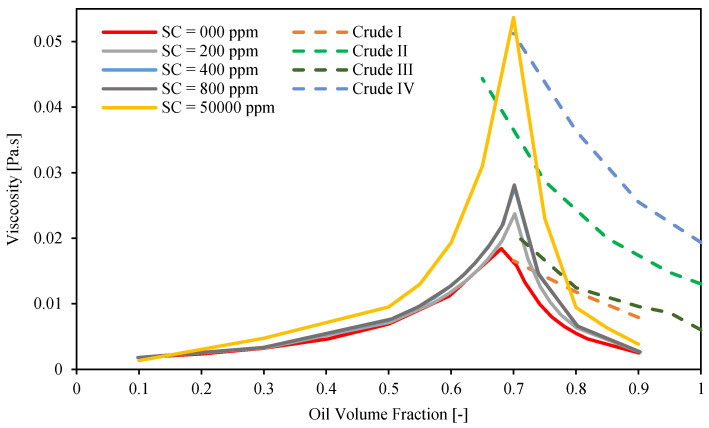
Comparison between predicted viscosities of different spiked concentration in Exxsol D60 salty-water fluid system and four different crude oil emulsions.

Figure 13 shows a comparison between viscosities of mixtures of Exxsol D60 and salty water spiked with different crude concentrations, calculated with the tuned Pal and Rhodes models [39] and experimental data for four crude oil emulsions (Crude I, Crude II, Crude III, and Crude IV) and salt water, as reported by Plasencia et al. [34]. Since the viscosities of Crude I and Crude III are lower than those of the other crude oils, their emulsion viscosities are also lower. The dispersion flow of Crude IV exhibits the highest emulsion viscosity.

Figure 13 shows that by adding modest amounts of crude to Exxsol D60, it is possible to mimic the rheology behavior of real crude emulsions in the vicinity of the inversion point. For example, Exxsol D60 spiked with 200 ppm shows similar viscosity at inversion point to emulsions of Crude I and crude III, respectively. On the other hand, to achieve viscosities similar to the ones exhibited by emulsions of heavier crudes such as Crude II and Crude IV requires using higher spiking concentrations in Exxsol D60.

As an example, to reach the same viscosity of the Crude IV dispersed flow at inversion point, 50,000 ppm of crude spiking is required. Emulsion viscosity differences between spiked solutions and real crude samples are high at conditions with high oil volume fractions (>0.75). This is because the emulsion viscosity is approaching the pure-oil-phase viscosity at these conditions, and the viscosity difference between Exxsol D60 and real crude oil is high.

### 3.4. Bottle Separation Tests

**Figure 14:**
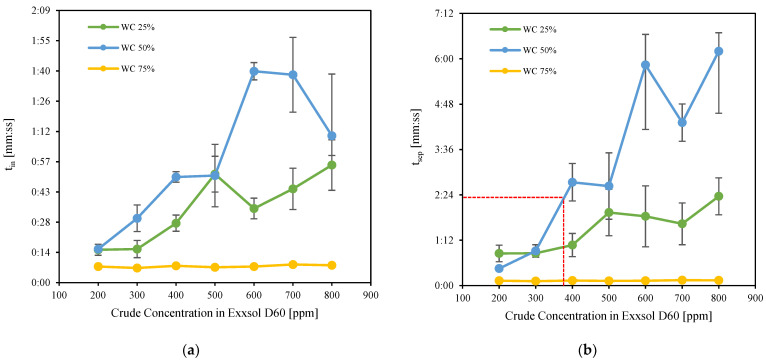
(**a**) Required time for fixed interface, t_in_, (**b**) Required time for phase separation, t_sep_ versus concentration of crude oil in the spiked Exxsol D60.

Figure 14 shows separation times for three water fractions (25%, 50%, 75%) as well as several spiked oil concentrations. With the rise in crude oil concentration in Exxsol D60, both t_in_ and t_sep_ increase dramatically in comparison to pure Exxsol D60 and salt water for water cuts of 50% and 25%. t_in_ and t_sep_ are higher at WC 50% than at WC 25% for the majority of crude oil concentrations. t_in_ and t_sep_ versus crude concentration have a non-monotonic relationship. There was no discernible difference in t_in_ or t_sep_ when the WC was set to 75%.

**Figure 15:**
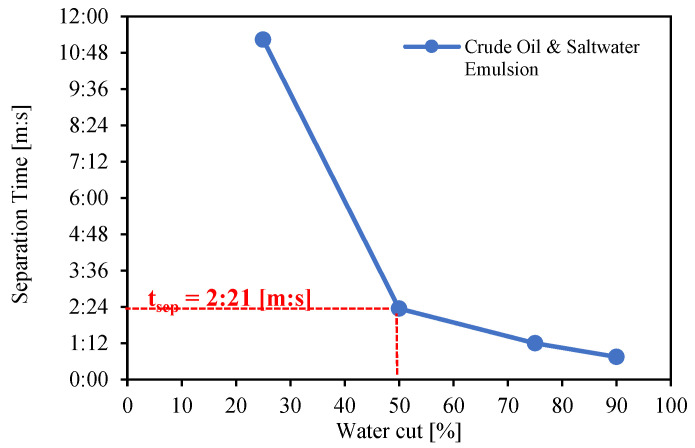
Effect of water cut on separation time of crude oil and salty-water mixture.

The separation time for pure crude oil was determined and is depicted in Figure 15 using four different water cuts of salt water. These tests were conducted at a temperature of 60 °C. The results reveal that as the water cut is reduced, separation time increases. When the water cut is reduced from 50% to 25%, the separation time increases from 2 min and 21 s to 11 min and 38 s. The produced emulsion layer is more stable with a 25% water cut, according to this trend.

**Figure 16:**
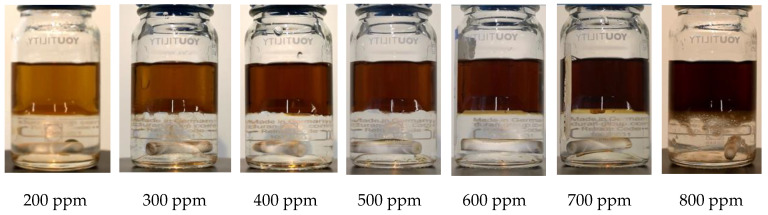
Visibility Test—Mixture of salt water with Exxsol D60 spiked with seven different amounts of crude oil.

Based on these results, it is possible to obtain similar separation times for the spiked oil–salty water mixture than for the original crude oil–salty-water mixture, depending on the spiking concentration and the water cut. For example, separation time for crude oil with a 50% water cut is 2 min and 21 s. The same separation time can be achieved with Exxsol D60 with a spiked concentration of close to 400 ppm at 50% water cut. Figure 13 shows photos of bottles with oil and water, for several spiking concentrations. Adding 400 ppm crude spiking to Exxsol D60 during the separation performance test did not affect significantly the visibility of the mixture in a transparent container, as is shown in Figure 16.

### 3.5. Droplet Size Distribution

In this section, the distribution of droplet size at different conditions of dispersed flow in the pipe is investigated to evaluate the effect of different flow parameters like water cut, crude spiking concentration, and mixture velocity. Additionally, the droplet size distribution of the dispersed flow of four real crude oils reported in the literature by Plasencia et al. [34] are compared with mixtures of Exxsol D60 and salt water with different spiking concentrations.

**Figure 17:**
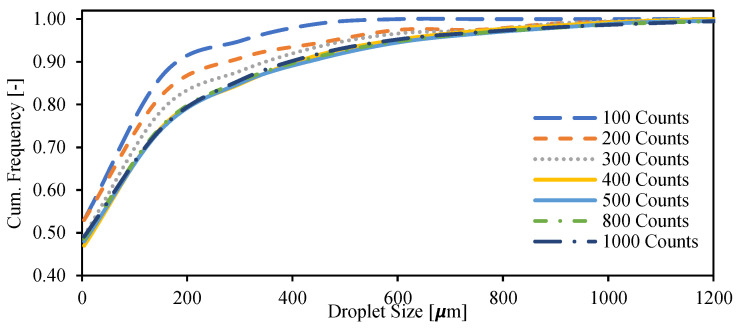
Primary counting of droplet size distribution of dispersed flow of Exxsol D60 and salt water in pipe.

The droplet distribution was determined by performing a manual count and measurement of droplets on the images. There are multiple images for each flow condition. After fully counting an image, the counting is continued to the following image. The process is repeated until the desired count number is reached. Figure 17 shows the droplet size cumulative distribution for a case (with 200 ppm spiking concentration, U_mix_ = 0.66 m/s and water cut 90%) using different numbers of droplet counts. It was found that at least 400 droplets should be counted for the results to be independent of the number of counts. In the following sections, 1000 droplets were counted. The same approach is applied for other conditions with different water cuts, crude spiking concentrations and mixture velocities. The results were similar to the one illustrated in Figure 17.

**Figure 18:**
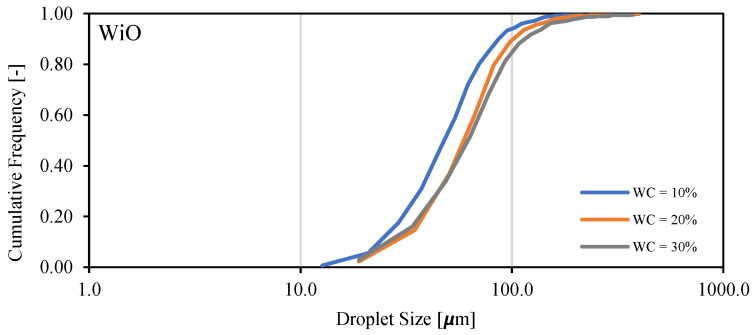
Droplet size distribution of Exxsol D60 spiked with 400 ppm crude and salty-water WiO emulsion at 0.66 m/s mixture velocity.

**Figure 19:**
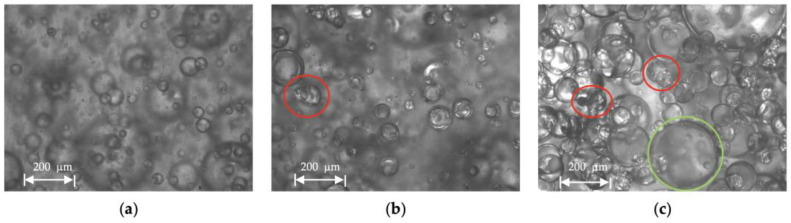
Microscopic pictures from Exssol D60 spiked with 400 ppm crude and salty-water WiO emulsion at 0.66 m/s mixture velocity with (**a**) 10%, (**b**) 20% and (**c**) 30% water cut.

Droplet size distribution of a water-in-oil emulsion of Exxsol D60 with 400 ppm crude spiking and salt water at 0.66 m/s mixture velocity is displayed in Figure 18 for water cuts of 10, 20 and 30%. The higher the water fraction, the more the curve shifts to the right (i.e., larger water droplet sizes). This is observed in Figure 19: at higher water cuts, the droplet coalescence is more noticeable (red circles in Figure 19b,c). The droplet coalescence leads to having larger droplets and then creating a continuous phase of water where the finally inversion point happens, and the dispersed flow becomes an oil-in-water emulsion. At a water cut equal to 30%, oil-in-water emulsion in continuous oil phase is visible (green circle in Figure 19c).

**Figure 20:**
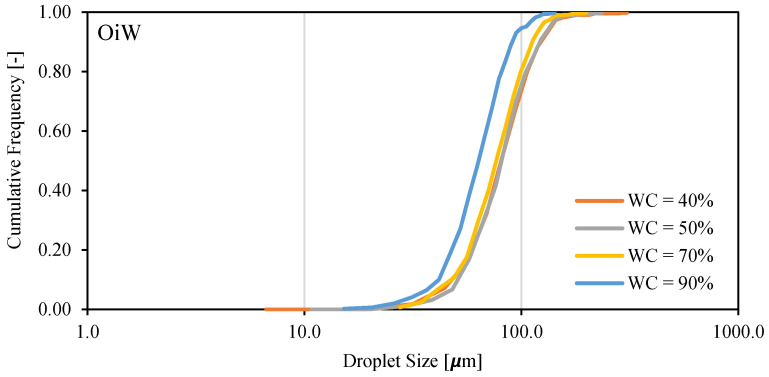
Droplet size distribution of Exxsol D60 spiked with 400 ppm crude and salty-water OiW emulsion at 0.66 m/s mixture velocity.

**Figure 21:**
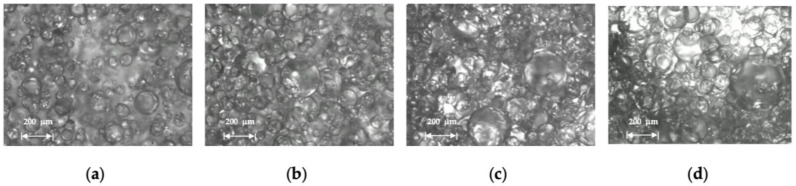
Microscopic pictures from Exxsol D60 spiked with 400 ppm crude and salty-water OiW emulsion at 0.66 m/s mixture velocity with (**a**) 90%, (**b**) 70%, (**c**) 50% and (**d**) 40% water cut.

In Figure 20, we observe that decreasing the water cut from 90 to 40% results in larger oil droplet sizes in the OiW emulsion. This change is more pronounced when the water cut changes from 90 to 70%. A high-volume fraction of the dispersed phase helps droplet coalescence to create larger droplets. The changes in droplet size are appreciable in Figure 21.

**Figure 22:**
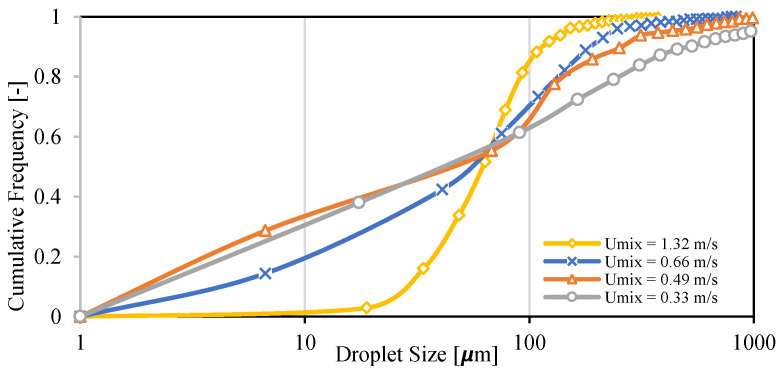
Droplet size distribution of Exxsol D60 spiked with 300 ppm crude and salty-water emulsion with 30% water cut at different mixture velocities.

**Figure 23:**
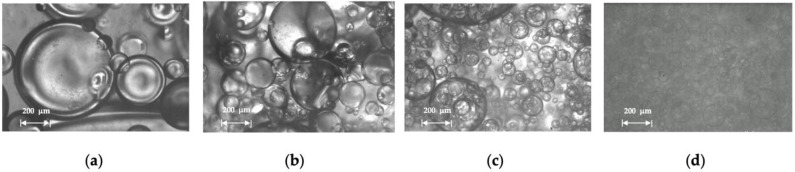
Microscopic pictures from Exxsol D60 spiked with 300 ppm crude and salty-water emulsion with 30% water cut at (**a**) 0.33, (**b**) 0.49, (**c**) 0.66 and (**d**) 1.32 m/s mixture velocities.

Droplet size distribution of dispersed flow of Exxsol D60 with 300 ppm crude spiking and salty water with 30% water cut at four different mixture velocities 0.33, 0.49, 0.66 and 1.32 m/s are displayed in Figure 22. This figure shows the effect of mixture velocity on the quality of dispersion flow. Increasing the mixture velocity results in higher shear rate and smaller droplet sizes. The flow condition with the highest mixture velocity, 1.32 m/s, exhibits the smallest droplet size distribution. This fact is visible in the microscopic pictures from the dispersed flow (Figure 23).

Since comparison of various flow conditions using droplet-size-distribution curves can be challenging, a weighted-mean diameter, which is less sensitive to very small and large droplets, is employed. The Sauter mean diameter d_32_ was computed as follows:

(4) $$d_{32}=\frac{\sum n_{i}d_{i}^{3}}{\sum n_{i}d_{i}^{2}},$$

**Figure 24:**
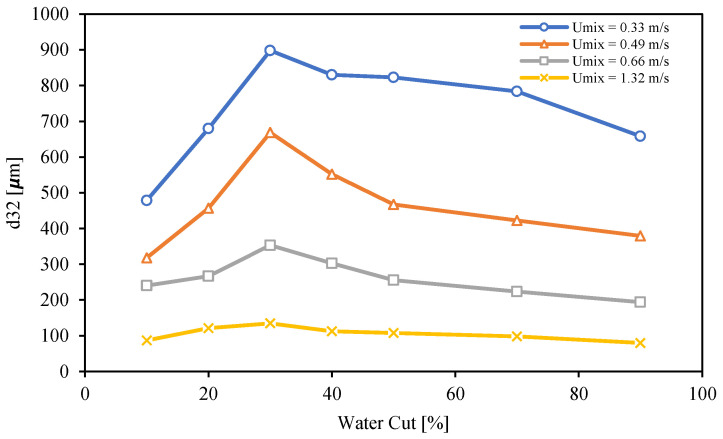
Sauter mean diameter of Exxsol D60 spiked with 300 ppm crude and salty-water emulsion at different mixture velocities and water cuts.

The Sauter mean diameters of dispersed flow of Exxsol D60 with 300 ppm crude spiking and salty water for range of water cuts from 10 to 90% and four different mixture velocities, 0.33, 0.49, 0.66 and 1.32 m/s are shown in Figure 24. Generally, and as it was discussed earlier, higher mixture velocities create a higher shear rate and cause smaller droplets. Figure 24 confirms this fact for all water cuts. Also, the Sauter mean diameter at a water cut of 30% is the highest value compared to the rest of the water cuts. This shows that, in the vicinity of the inversion point, the droplets of the dispersed phase have the highest diameter. According to Figure 7, a flow condition with 30% water cut is considered as OiW emulsion just for the lowest mixture velocity, 0.33 m/s; meanwhile, for other mixture velocities, the visualized droplets at the conditions with water cut of 30% are dispersed water droplets in continuous oil phase.

**Figure 25:**
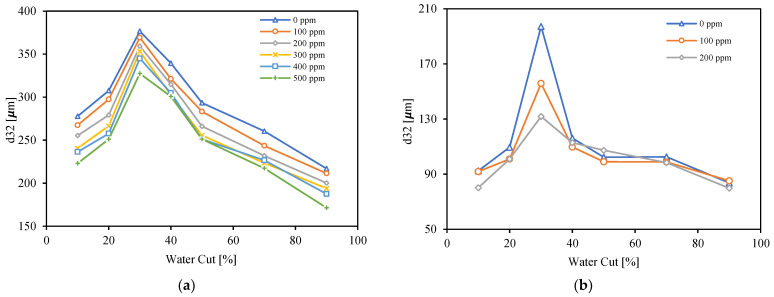
Sauter mean diameter of Exxsol D60 spiked with crude and salty-water emulsion at different spiked concentrations and water cuts. (**a**) 0.66 m/s, (**b**) 1.32 m/s.

Adding crude spiking brings surface-active agents like asphaltene molecules onto the droplet surface. This prevents quick coalescence between droplets and promotes the formation of a stable emulsion phase. After applying the same shear rate, small droplets tend to stay longer as a dispersion. This is observed in Figure 25a,b, where crude spiking results in smaller droplets in both mixture velocities 0.66 (Figure 25a) and 1.32 m/s (Figure 25b). However, at a mixture velocity equal to 1.32 m/s (Figure 25b) there are no significant changes in Sauter mean diameters with spiking, except at a water cut equal to 30%.

**Figure 26:**
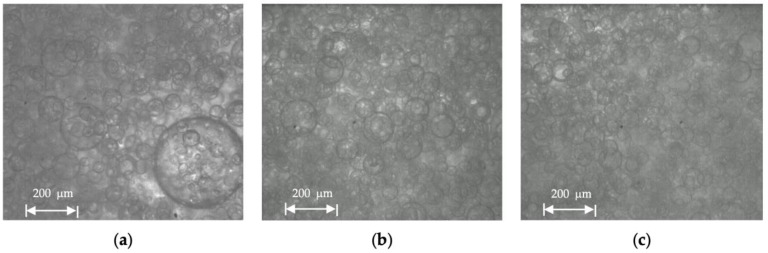
Microscopic pictures from Exxsol D60 spiked with crude and salty-water emulsion with 30% water cut at 1.32 m/s mixture velocity with: (**a**) no crude spiking, (**b**) 100 ppm and (**c**) 200 ppm crude spiking.

Microscopic pictures from dispersed flow with 30% water cut and at 1.32 m/s mixture velocity and different crude spiking 0, 100 and 200 ppm show that adding crude spiking results in smaller droplet sizes (Figure 26a–c). Overall, with the same dispersed volume fraction, WiO emulsion has smaller droplets than OiW emulsion (e.g., 10% dispersed volume fraction).

**Figure 27:**
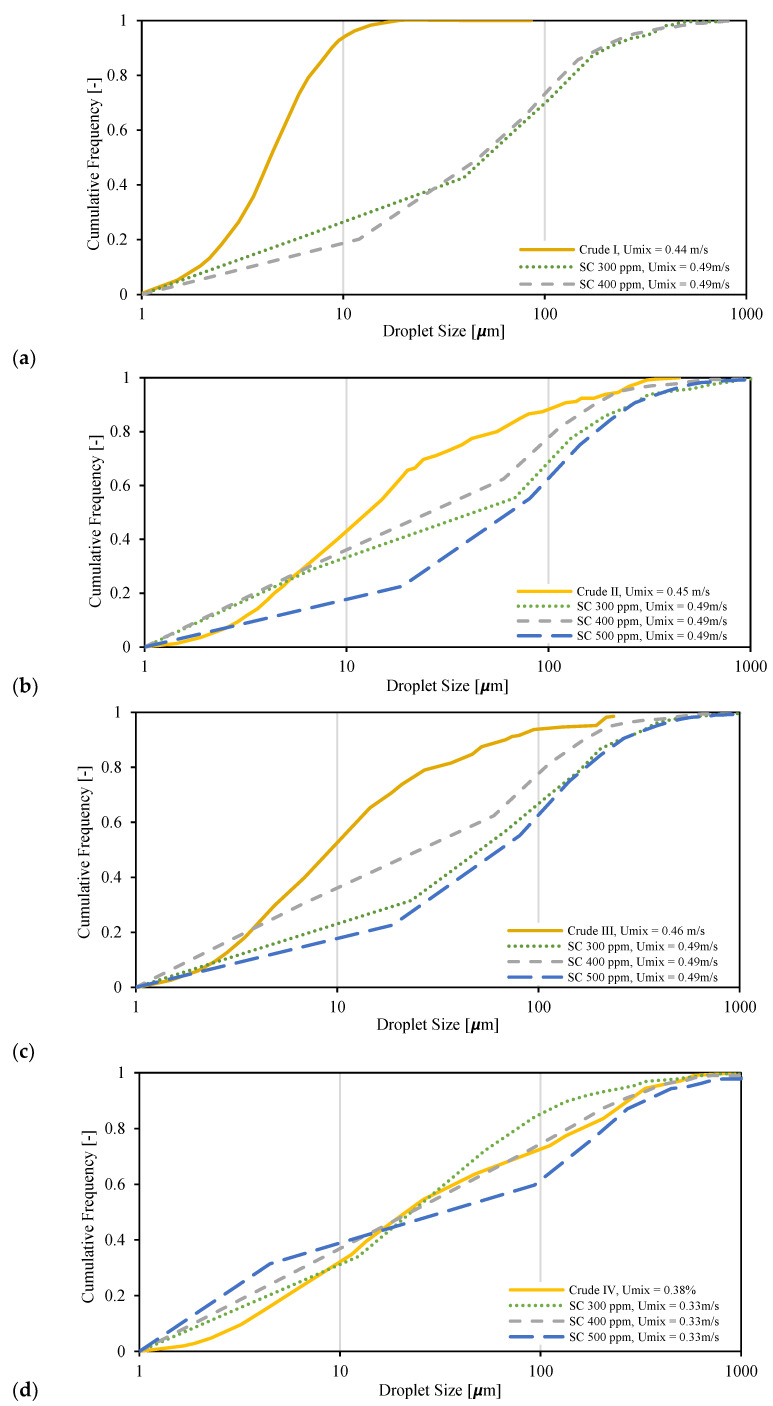
Comparison between droplet size distribution of different spiked concentration in Exxsol D60 salty-water fluid system and four different crude oil emulsions: (**a**) crude I with 10% water cut, (**b**) crude II with 30% water cut, (**c**) crude III with 30% water cut, (**d**) crude IV with 10% water cut.

Finally, the droplet size distributions of four real crude oils and salty-water emulsions taken from the literature [34] are compared to the results of Exxsol D60 spiked with crude and salty water (Figure 27). The droplet size distribution of Crude I was very different from curves of spiked Exxsol D60 + salty-water mixtures (Figure 27a); meanwhile, for other crude oils it is possible to achieve a similar droplet size distribution by adding a small amount of crude spiking. For example, with 10% water cut, a mixture with 400 ppm crude spiking has a roughly similar droplet size distribution to that of Crude IV (Figure 27d).

## 4. Conclusions

The experimental investigation delves into various attributes of dispersed pipe flow involving Exxsol D60 enriched with crude oil and salty water. Parameters like inversion point, rheology model, viscosity, separation time, and droplet size distribution are examined. Furthermore, dispersion data from assorted mixtures of crude oil and salty water sourced from literature were juxtaposed with this data to ascertain the requisite concentration of crude oil spiking for emulating the pipe flow of authentic crude–salty-water dispersion.

For the Exxsol D60 and salty-water fluid system, the phase inversion arises at approximately 0.7 oil volume fraction (equivalent to a 30% water cut). Below this inversion point, multiple emulsions and droplet coalescence are observable. Following the assessment of various rheology models, the Pal and Rhodes model emerged as the most suitable for predicting viscosity in the studied fluid system. Modest crude spiking yields an emulsion viscosity akin to that of light crude and salty-water emulsions. However, achieving comparable emulsion viscosities for viscous crude and salty water demands substantial crude spiking (around 50,000 ppm).

Experiments indicate that introducing roughly 400 ppm of crude spiking effectively prolongs the separation time of Exxsol D60 and salty-water mixture at room temperature to match that of a crude–salty-water mixture at 60 °C. An augmented presence of surface-active molecules due to crude spiking enables the prolonged retention of small droplets within the mixture, resulting in enhanced stability. WiO emulsions exhibit smaller droplets compared to OiW emulsions at the same dispersed volume fraction. Comparative analysis of droplet size distributions between emulsions of genuine crude oils with salty water and the Exxsol D60 fluid system spiked with oil and salty water underscores the feasibility of achieving a similar droplet size distribution through spiking.

## Figures and Tables

**Table 1:** Literature review.

Reference with Year	Examples of Stabilizer
Brown et al. (1972) [28]	Kerosene and salty water
Calabrese et al. (1986) [29]	Silicon oil and water system
Boxall et al. (2010) [30]	Crude and mineral oil mixes to change the viscosity w/o altering the interfacial tension
Rodionova et al. (2014) [27]	Primol 352 and Exxsol D60 mixtures with or without SPAN^®^ 83
Keleşoğlu et al. (2015) [26]	Aerosil^®^ R104 and myristic acids
Fossen et al. (2016) [31]	Primol 352 and polyisobutylene mixture, Primol 352 and polyisobutylene mixture
Ahmadi (2021) [32]	Crude oil and Calcium Oxide Nanoparticles
Mansouri et al. (2022) [33]	Crude oil and zirconia- zinc-copper nanocomposite

**Table 2:** Fluid properties.

Fluid	Density [kg/m^3^]	Viscosity [cP]
Distilled Water w/wt% 3.4 NaCl	1020.7	0.99
Exxsol D60 w/0.015 g/L Oil Red O	787.45	1.31

**Table 3:** Crude oil properties at 15 °C.

Viscosity [cP]	Density [kg/m^3^]	TAN [mg KOH/g]	TBN [mg KOH/g]	Asphaltenes Content
24.01	939.3	2.15	2.81	2.5 wt% hexane insoluble

**Table 4:** Crude oil properties [34].

Crude	Viscosity [cP]	Density [kg/m^3^]	TAN [mg KOH/g]	Asphaltene Content [wt%]	Interfacial Tension [mN/m]	Temperature [°C]
I	7.5	865	1.7	0.6	4.5	60
II	15.9	885	1.4	7.3	12.5	70
III	7.1	834	0.5	6.9	12.3	70
IV	23.5	901	4.3	2.8	11.1	70

**Table 5:** Experimental campaign test matrix.

Mixture Velocity [m/s]	Oil Volume Fractions [-]	Spiking Concentration [ppm]
0.33	10/20/30/40/50/55/57/60/62/64/ 68/70/72/74/78/80/90	300/400/500/600/700/800/900
0.49	10/20/30/40/50/55/57/60/62/64/ 68/70/72/74/78/80/90	300/400/500/600/700/800/900
0.66	10/20/30/40/50/55/57/60/62/64/ 68/70/72/74/78/80/90	0/100/200/300/400/500/600/700/800/900/50,000
1.32	10/20/30/40/50/55/57/60/62/64/ 68/70/72/74/78/80/90	0/100/200

**Table 6:** Test matrix for bottle separation tests.

Crude Concentration [ppm]	WC [%]
200/300/400/500/600/700/800	25
200/300/400/500/600/700/800	50
200/300/400/500/600/700/800	75

**Table 7:** Inversion point at different mixture velocities.

Mixture Velocity [m/s]	Oil Volume Fraction at Inversion Point [-]
0.33	71
0.49	70
0.66	69
1.32	67

**Table 8:** Rheology models of oil–water emulsion from the literature.

Reference with Year	Model Equation	Liquids	Equation Considerations
Eilers [36] (1941)	$\eta_{r}={\left(1+\frac{1.25\Omega }{1-K_{E}\Omega }\right)}^{2}$	Bayol-35 with 0.5% (*v*/*v*) Triton x-100	$1.28<K_{E}<1.30$ 0.19 < Ω < 0.68
Roscoe [37] (1952)	$\eta_{r}={\left(1-\Omega \right)}^{-2.5}$ $\eta_{r}={\left(1-1.35\Omega \right)}^{-2.5}$	Shell vitrea-220 with 0.5% (*v*/*v*) Triton x-100	0.3 < Ω < 0.69
Taylor [38] (1932)	$\eta_{r}=1+2.5\left(\frac{\mu_{d}+0.4\mu_{c}}{\mu_{d}+\mu_{c}}\right)\Omega$	CCl4/Bayol mix. (26.55% (*v*/*v*) CCl4) with 0.5% (*v*/*v*) Triton x-100	$\mu_{d}$ and $\mu_{c}$ are the dispersed-phase and continuous-phase viscosities, respectively.
Pal and Rhodes [39] (1989)	$\eta_{r}={\left(1+\frac{\frac{\Omega }{K_{PR}}}{1.1884-\frac{\Omega }{K_{PR}}}\right)}^{2.5}$	Diesel with 2% (*v*/*v*) Span 85	Ω < 0.74
Homogenous model	$\eta_{e}=\Omega \mu_{d}+\left(1-\Omega \right)\mu_{c}$	---	---

## Data Availability

Not applicable.

## Abbreviations

*D*	Pipe inner diameter [m]
*K*	Viscosity model constant [-]
*L*	Pipe length [m]
*Re*	Reynolds Number [-]
*T*	Temperature [°C]
*U*	Mixture velocity [m/s]
*e*	Pipe roughness [mm]
*f*	Friction factor [-]
ρ	Density [kg/m^3^]
*t*	Time [s]
𝜇	Viscosity [cP]
𝛾	Shear rate [s^−1^]
*d*	Droplet size distribution [μm]
𝜂	Viscosity [cP]
τ	Shear stress [Pa]
Ω	Volume fraction [-]
ΔP	Pressure drops [Pa]
**Subscript**	
*PR*	Pal and Rhodes model
*c*	continuous phase
*d*	dispersed phase
*e*	emulsion
*eff*	effective
*in*	interface stabilization
*m*	mixture
*r*	relative
*sep*	separation

## References

[1] Schümann H., Tutkun M., Nydal O.J. (2016). Experimental study of dispersed oil-water flow in a horizontal pipe with enhanced inlet mixing, Part 2: In-Situ droplet measurements. J. Pet. Sci. Eng..

[2] Lim J., Wong S., Law M., Samyudia Y., Dol S. (2015). A review on the effects of emulsions on flow behaviours and common factors affecting the stability of emulsions. J. Appl. Sci..

[3] Chen G., Tao D. (2005). An experimental study of stability of oil–water emulsion. Fuel Process. Technol..

[4] Yarranton H.W., Sztukowski D.M., Urrutia P. (2007). Effect of interfacial rheology on model emulsion coalescence: I. Interfacial rheology. J. Colloid Interface Sci..

[5] Sjoblom J. (2001). Encyclopedic Handbook of Emulsion Technology.

[6] Bibette J., Leal-Calderon F., Schmitt V., Poulin P. (2003). Emulsion Science: Basic Principles. An Overview.

[7] Speight J.G. (2006). The Chemistry and Technology of Petroleum.

[8] Ancheyta J., Speight J.G. (2007). Heavy Oils and Residua. Hydroprocessing of Heavy Oils and Residua.

[9] Asaadian H., Soulgani B.S., Karimi A. (2017). An experimental study on electrical effect on asphaltene deposition. Pet. Sci. Technol..

[10] Ahmadi P., Asaadian H., Kord S., Khadivi A. (2019). Investigation of the simultaneous chemicals influences to promote oil-in-water emulsions stability during enhanced oil recovery applications. J. Mol. Liq..

[11] Sjöblom J., Aske N., Auflem I.H., Brandal Ø., Havre T.E., Sæther Ø., Westvik A., Johnsen E.E., Kallevik H. (2003). Our current understanding of water-in-crude oil emulsions: Recent characterization techniques and high pressure performance. Adv. Colloid Interface Sci..

[12] Aske N., Kallevik H., Sjöblom J. (2001). Determination of saturate, aromatic, resin, and asphaltenic (SARA) components in crude oils by means of infrared and near-infrared spectroscopy. Energy Fuels.

[13] Belt R., Duret E., Larrey D., Djoric B., Kalali S. (2011). Comparison of commercial multiphase flow simulators with experimental and field databases. Proceedings of the 15th International Conference on Multiphase Production Technology.

[14] Lakehal D. (2013). Advanced simulation of transient multiphase flow & flow assurance in the oil & gas industry. Can. J. Chem. Eng..

[15] Lee R.F. (1999). Agents which promote and stabilize water-in-oil emulsions. Spill Sci. Technol. Bull..

[16] Langevin D., Poteau S., Hénaut I., Argillier J.F. (2004). Crude oil emulsion properties and their application to heavy oil transportation. Oil Gas Sci. Technol..

[17] Ahmadi P., Asaadian H., Khadivi A., Kord S. (2019). A new approach for determination of carbonate rock electrostatic double layer variation towards wettability alteration. J. Mol. Liq..

[18] Moghadasi R., Kord S., Moghadasi J., Asaadian H. (2018). On the Effects of Salinity, Asphaltenes and Resins on Interfacial Tension: Applications to Low Salinity Water Injection. Proceedings of the 80th EAGE Conference and Exhibition 2018.

[19] Mansouri M., Ahmadi Y. (2022). Applications of zeolite-zirconia-copper nanocomposites as a new asphaltene inhibitor for improving permeability reduction during CO_2_ flooding. Sci. Rep..

[20] Ahmadi Y., Aminshahidy B. (2020). Inhibition of asphaltene precipitation by hydrophobic CaO and SiO_2_ nanoparticles during natural depletion and CO_2_ tests. Int. J. Oil Gas Coal Technol..

[21] Schramm L.L. (1992). Fundamentals and applications in the petroleum Industry. Adv. Chem..

[22] Pal R. (2006). Rheology of Particulate Dispersions and Composites.

[23] Schuster D. (1996). Encyclopedia of Emulsion Technology.

[24] Gafonova O.V., Yarranton H.W. (2001). The stabilization of water-in-hydrocarbon emulsions by asphaltenes and resins. J. Colloid Interface Sci..

[25] Kilpatrick P.K. (2012). Water-in-crude oil emulsion stabilization: Review and unanswered questions. Energy Fuels.

[26] Keleşoğlu S., Rodionova G., Pettersen B.H., Foss M., Sjöblom J. (2015). Preparation and characterization of reference fluids to mimic flow properties of crude oil emulsions (w/o). J. Dispers. Sci. Technol..

[27] Rodionova G., Pettersen B., Kelesoğlu S., Sjöblom J. (2014). Preparation and characterization of reference fluid mimicking behavior of North Sea heavy crude oil. Fuel.

[28] Brown D.E., Pitt K. (1972). Drop size distribution of stirred non-coalescing liquid—Liquid system. Chem. Eng. Sci..

[29] Calabrese R.V., Chang T.P.K., Dang P.T. (1986). Drop breakup in turbulent stirred-tank contactors. Part I: Effect of dispersed-phase viscosity. AIChE J..

[30] Boxall J.A., Koh C.A., Sloan E.D., Sum A.K., Wu D.T. (2010). Measurement and calibration of droplet size distributions in water-in-oil emulsions by particle video microscope and a focused beam reflectance method. Ind. Eng. Chem. Res..

[31] Fossen M., Schümann H. (2017). Experimental study of the relative effect of pressure drop and flow rate on the droplet size downstream of a pipe restriction. J. Dispers. Sci. Technol..

[32] Ahmadi Y. (2021). Improving fluid flow through low permeability reservoir in the presence of nanoparticles: An experimental core flooding WAG tests. Iran. J. Oil Gas Sci. Technol..

[33] Mansouri M., Ahmadi Y., Jafarbeigi E. (2022). Introducing a new method of using nanocomposites for preventing asphaltene aggregation during real static and dynamic natural depletion tests. Energy Sources Part A Recovery Util. Environ. Eff..

[34] Plasencia J., Pettersen B., Nydal O.J. (2013). Pipe flow of water-in-crude oil emulsions: Effective viscosity, inversion point and droplet size distribution. J. Pet. Sci. Eng..

[35] Ronningsen H.P. (1995). Correlations for predicting viscosity of W/O-emulsions based on North Sea crude oils. Proceedings of the SPE International Symposium on Oilfield Chemistry.

[36] Eilers V.H. (1941). Die viskosität von emulsionen hochviskoser stoffe als funktion der konzentration. Kolloid Z..

[37] Roscoe R. (1962). The end correction for rotation viscometers. Br. J. Appl. Phys..

[38] Taylor G.I. (1932). The viscosity of a fluid containing small drops of another fluid. In Proceedings of the Royal Society of London. Ser. A Contain. Pap. A Math. Phys. Character.

[39] Pal R., Rhodes E. (1989). Viscosity/concentration relationships for emulsions. J. Rheol..

